# Codevelopment and evaluation of a multicomponent intervention to improve HPV vaccination in France: Aurélie Bocquier

**DOI:** 10.1093/eurpub/ckac131.340

**Published:** 2022-10-25

**Authors:** A Bocquier, S Bonnay, S Bruel, K Chevreul, A Gagneux-Brunon, A Gauchet, B Giraudeau, AS Le Duc-Banaszuc, JE Mueller, N Thilly

**Affiliations:** 1 APEMAC, Université de Lorraine, Nancy, France; 2 Département de Médecine Générale, Université Paris, Paris, France; 3 HESPER EA7425, Saint-Etienne-Lyon University, Saint-Etienne, France; 4 CIC-INSERM 1408, University Hospital of Saint-Etienne, Saint-Etienne, France; 5 ECEVE UMR 1123, Université de Paris, Paris, France; 6 URC Eco Ile-de-France, Hôpital Robert Debré, Paris, France; 7 Team GIMAP, CHU de Saint-Etienne, Saint-Etienne, France; 8 LIP/PC2S, University Savoie Mont Blanc, Chambéry, France; 9 SPHERE U1246, Université de Tours, Université de Nantes, INSERM, Tours, France; 10 INSERM CIC 1415, CHRU de Tours, Tours, France; 11 CRCDC Pays de la Loire, Angers, France; 12 Unité Epidémiologie des Maladies Émergentes, Institut Pasteur, Paris, France; 13 EHESP French School of Public Health, Rennes, France; 14 Méthodologie, Promotion, Investigation, Université de Lorraine, CHRU-Nancy, Nancy, France

## Abstract

**Background:**

HPV vaccine coverage (VC) in France has always been lower than in most high-income countries. The French authorities launched in 2018 the PrevHPV national research program aimed at codeveloping with stakeholders and evaluating the impact of a multicomponent intervention to improve HPV VC among French adolescents.

**Methods:**

We identified three components to address main barriers to HPV vaccination in France: adolescents’ and parents’ education and motivation (component 1); general practitioners (GPs)’ training (component 2); and access to vaccination at school (component 3). We developed the intervention using the UK Medical Research Council framework for developing complex interventions as a guide. We used (i) findings from published evidence; (ii) primary data on knowledge, attitudes, behavior and preferences collected through a mixed methods approach (quantitative/qualitative studies, discrete choice experiment); (iii) the advice of stakeholders (e.g., adolescents, parents, school nurses, GPs) involved in working groups. We will evaluate the effectiveness, efficiency and implementation of the components (applied alone or in combination) through a pragmatic cluster randomized controlled trial. The primary endpoint is the HPV VC (≥ 1 dose) among adolescents aged 11-14 years, 2 months after the end of the intervention, at the municipality level.

**Results:**

Primary data highlighted the need to improve adolescents, parents and school staff knowledge on HPV and to help GPs communicate with patients on this topic. They provided guidance on the most effective communication contents. For each component, we codeveloped tools with a participatory approach (e.g., eHealth tools for adolescents, a decision aid tool for GPs). The trial will end in June 2022; 90 middle schools (i.e., about 40,000 adolescents) and 46 GPs accepted to participate.

**Conclusions:**

Should the intervention prove effective, results from the implementation evaluation will help us refine it before scaling it up.

**Key messages:**

• The PrevHPV study is supported by the French health authorities and conducted by a multidisciplinary consortium to tackle a long-lasting public health concern in France.

• It will add to the small number of studies that compared the effectiveness of various strategies to promote HPV vaccination and will provide key results on cost-effectiveness and implementation.

